# Explaining the Black-White Disparity in Preterm Birth: A Consensus Statement From a Multi-Disciplinary Scientific Work Group Convened by the March of Dimes

**DOI:** 10.3389/frph.2021.684207

**Published:** 2021-09-02

**Authors:** Paula Braveman, Tyan Parker Dominguez, Wylie Burke, Siobhan M. Dolan, David K. Stevenson, Fleda Mask Jackson, James W. Collins, Deborah A. Driscoll, Terinney Haley, Julia Acker, Gary M. Shaw, Edward R. B. McCabe, William W. Hay, Kent Thornburg, Dolores Acevedo-Garcia, José F. Cordero, Paul H. Wise, Gina Legaz, Kweli Rashied-Henry, Jordana Frost, Sarah Verbiest, Lisa Waddell

**Affiliations:** ^1^School of Medicine, University of California, San Francisco, San Francisco, CA, United States; ^2^USC Suzanne Dworak-Peck School of Social Work, University of Southern California, Los Angeles, CA, United States; ^3^University of Washington School of Medicine, Seattle, WA, United States; ^4^Albert Einstein College of Medicine, New York, NY, United States; ^5^Stanford University School of Medicine, Stanford, CA, United States; ^6^Majaica LLC, Atlanta, GA, United States; ^7^Northwestern University School of Medicine, Chicago, IL, United States; ^8^University of Pennsylvania School of Medicine, Philadelphia, PA, United States; ^9^David Geffen School of Medicine at University of California, Los Angeles, CA, United States; ^10^University of Colorado, Denver, CO, United States; ^11^School of Medicine, Oregon State University, Portland, OR, United States; ^12^Brandeis University School for Social Policy and Management, Waltham, MA, United States; ^13^University of Georgia College of Public Health, Athens, GA, United States; ^14^March of Dimes, White Plains, NY, United States; ^15^University of North Carolina at Chapel Hill, Chapel Hill, NC, United States

**Keywords:** preterm birth, racism, health disparities, maternal/infant health, stress, birth outcomes, social determinants of health, racial health disparities

## Abstract

In 2017–2019, the March of Dimes convened a workgroup with biomedical, clinical, and epidemiologic expertise to review knowledge of the causes of the persistent Black-White disparity in preterm birth (PTB). Multiple databases were searched to identify hypothesized causes examined in peer-reviewed literature, 33 hypothesized causes were reviewed for whether they plausibly affect PTB and either occur more/less frequently and/or have a larger/smaller effect size among Black women vs. White women. While definitive proof is lacking for most potential causes, most are biologically plausible. No single downstream or midstream factor explains the disparity or its social patterning, however, many likely play limited roles, e.g., while genetic factors likely contribute to PTB, they explain at most a small fraction of the disparity. Research links most hypothesized midstream causes, including socioeconomic factors and stress, with the disparity through their influence on the hypothesized downstream factors. Socioeconomic factors alone cannot explain the disparity's social patterning. Chronic stress could affect PTB through neuroendocrine and immune mechanisms leading to inflammation and immune dysfunction, stress could alter a woman's microbiota, immune response to infection, chronic disease risks, and behaviors, and trigger epigenetic changes influencing PTB risk. As an upstream factor, racism in multiple forms has repeatedly been linked with the plausible midstream/downstream factors, including socioeconomic disadvantage, stress, and toxic exposures. Racism is the only factor identified that directly or indirectly could explain the racial disparities in the plausible midstream/downstream causes and the observed social patterning. Historical and contemporary systemic racism can explain the racial disparities in socioeconomic opportunities that differentially expose African Americans to lifelong financial stress and associated health-harming conditions. Segregation places Black women in stressful surroundings and exposes them to environmental hazards. Race-based discriminatory treatment is a pervasive stressor for Black women of all socioeconomic levels, considering both incidents and the constant vigilance needed to prepare oneself for potential incidents. Racism is a highly plausible, major upstream contributor to the Black-White disparity in PTB through multiple pathways and biological mechanisms. While much is unknown, existing knowledge and core values (equity, justice) support addressing racism in efforts to eliminate the racial disparity in PTB.

## Introduction

In 2017, the March of Dimes (MOD) (www.marchofdimes.org) launched an initiative to address the large and persistent racial disparities in preterm birth (PTB) in the United States. A multidisciplinary scientific workgroup was convened to conduct a state-of-the-science literature review of current knowledge of the causes of the Black-White disparity in PTB. This paper represents the consensus of the Workgroup's members.

Preterm birth (PTB, live birth < 37weeks) is the second leading cause of infant mortality in the United States (1) overall and the leading cause of infant mortality among African American/Black infants (2). Preterm infants are at higher risk for major adverse health outcomes in childhood (3–6) and adulthood (7–14).

While PTB has declined over the past century, African American/Black women consistently experience a rate approximately 1.5–1.6 times higher than that of Whites (15–17). Although hypotheses vary, most scientists agree that the causes of the Black-White disparity are complex and require further elucidation (18–22). This paper aims to critically review current knowledge about the Black-White disparity in PTB to inform efforts to eliminate the gap while improving birth outcomes overall. It is important to note that a cause of PTB is not necessarily a cause of the Black-White disparity in PTB. To be a cause of the disparity, a factor must not only influence PTB, it also must have a different prevalence and/or effect size among Black vs. White women.

## Methods

Research assistants searched the PubMed, ERIC, Scopus, Science Direct, and Google Scholar databases using preterm, premature, prematurity, and gestational age as search terms, and risk factors suggested by the Workgroup, aiming to include all factors that have received scientific attention. Peer-reviewed, English-language articles published in the past 10 years or classic/key papers addressing whether a given factor: (a) plausibly contributes to PTB, (b) occurs more/less frequently among Blacks, and/or (c) has a larger/smaller effect among Blacks were selected. Limited web-based material from scientifically trustworthy sources [e.g., Centers for Disease Control and Prevention (CDC) reports] also were included. The Workgroup Chair led a subcommittee in drafting an initial paper and subsequent revisions for members' review. Revisions continued until consensus was reached.

**Figure 1:**
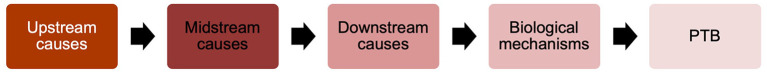
Potential causes of the Black-White disparity in PTB can be categorized by the hypothesized proximity of their effects on PTB.

As illustrated in Figure 1, based on the metaphor of a river flowing from its upstream source down to its downstream destination, we categorized potential causes as downstream, midstream or upstream, according to the proximity of their hypothesized effects to the outcome of interest (PTB). Downstream factors exert direct effects (e.g., they relatively directly trigger physiological mechanisms that result in PTB). Upstream factors have their effects further away from PTB, i.e., at or near the start of the causal chain, they may operate indirectly through midstream (intermediate) factors, which, in turn, affect downstream factors. Although interpretations have varied, the upstream/downstream theoretical framework has been widely used in public health to differentiate fundamental, underlying causes of ill health from the factors they set in motion (23).

## Findings From the Review, Including Comments on the Plausibility of Each Hypothesized Factor

Table 1 lists all of the factors examined, organized according to whether they act primarily downstream, midstream, or upstream. Some of the factors, however, could logically be grouped in more than one category. It also is important to note that many of the factors discussed below may interact with each other (and potentially with other factors not listed) in complex ways that can alter their primary effects. Such “syndemic” (24) effects may help explain difficulties researchers have encountered in elucidating the role of these factors in PTB.

**Table 1:** Summary table of factors that may influence the black-white disparity in preterm birth.

**Downstream factors hypothesized as causes of the Black-White disparity in preterm birth**	**Weight of the evidence that the factor contributes to the Black-White disparity in preterm birth**
Prenatal care	Plausible but existing literature generally does not support a role for quantitative measures of traditional prenatal care
Preconception care	Plausible contributing factor but little research available
Elective and indicated cesarean section	Plausible contributing factors but insufficient research
Substance use disorders	No published studies indicate that substance use explains the Black-White disparity in PTB
Diet/nutrition	Plausible but more robust research needed
Gestational weight gain (GWG)	Unclear whether excessive or inadequate GWG contribute to the disparity
Obesity	Black women's higher obesity rates could contribute but cannot explain the disparity among non-obese women
Inter-pregnancy intervals	Potential small contributor
Age	Not a plausible cause of the disparity
Gestational diabetes (GDM)	May contribute but its greater effect size is balanced by lower prevalence among Black women
Hypertensive disorders of pregnancy (HDP)	Likely important contributor
Pre-pregnancy (pre-existing) diabetes	Plausible but low prevalence among Black women
Pre-pregnancy (chronic) hypertension	Highly plausible but cannot explain PTB among women without this risk factor
Infection	Plausible but research inconclusive
Microbiota	Plausible
Neighborhood environmental exposures	Exposures to neighborhood social and physical hazards are highly plausible and potentially major contributors
Cold and heat	Plausible but too few studies
Genetic and epigenetic factors	Genetic factors are unlikely to play a major role in the disparity (vs. in PTB), epigenetic factors may be important
**Midstream Causes (which exert their effects through downstream factors)**	
Stress	Likely a major contributor through neuroendocrine mechanisms. More research needed on life-course (vs. pregnancy-only) stress
Depression	Unlikely to explain the disparity
Resilience	Evidence insufficient
Coping	Evidence is inconsistent and insufficient
Social support	Plausible but research is inconsistent and has focused on support during pregnancy alone
Midstream paternal factors	Plausible through multiple causal pathways including those involving stress and socioeconomic factors
Income and education	Plausible but relationships are complex due to racism
Childhood and lifelong socioeconomic status	Plausible but rarely measured
Wealth	Plausible but we did not identify literature on wealth and the PTB disparity
Educational quality	Plausible but we did not identify literature on educational quality and the PTB disparity
Neighborhood socioeconomic disadvantage	Highly plausible, considerable literature supports
**Upstream Causes**	
Racism in multiple forms and through multiple pathways and biological mechanisms	Highly plausible

*As noted elsewhere in this paper, a factor may influence PTB without influencing the racial disparity, unless it is more prevalent or has a stronger effect size among African American women (or women of European descent)*.

**DOWNSTREAM FACTORS** are hypothesized to directly influence the biological mechanisms that produce PTB.

### Prenatal Care

With rare exceptions, studies controlling for key maternal characteristics have not found associations between PTB and (quantitatively) inadequate prenatal care (e.g., late initiation, insufficient number of visits) (25–28). PTB rates have risen (16, 29, 30) and the racial gap in PTB has persisted (31) despite increased prenatal care usage and timely initiation overall and a narrowing of the Black-White gap in prenatal care (32). Some researchers, however, report better outcomes and smaller Black-White disparities for Black women with greater access to prenatal care (33, 34). Quality of care is another consideration. Black people receive lower quality medical services generally (35–37), and lower quality prenatal care specifically (e.g., less medical advice, fewer recommended treatments) (38–40), although not all studies have documented such differences (41, 42). The weight of evidence suggests that racial differences in traditional prenatal care are unlikely to explain the racial disparity in PTB (43, 44).

Group prenatal care, such as Centering Pregnancy (45) and Moms2Be (46), involves extended visits and more biopsychosocial support, including peer support, than traditional prenatal care. It has been associated with significantly reduced PTB and other favorable perinatal outcomes (e.g., greater pregnancy knowledge, lower stress, higher breastfeeding rates, less food insecurity, lower hospitalization costs, greater satisfaction with care, healthier behaviors) in observational (46–52), cohort (53–55) and randomized studies (56–58). Better pregnancy experiences (58) and outcomes (46, 56) in predominantly African American samples, and a smaller racial gap in PTB (55) have been linked to group prenatal care. Although literature reviews indicate that group care is a promising model, especially for higher risk groups like African Americans (59–62), more rigorous studies are needed to determine if group care can reduce PTB and the Black-White disparity in PTB, given inconsistent study results (49, 53, 54, 58, 63).

### Preconception Care

African-American women of reproductive age experience elevated rates of a variety of chronic conditions that can enhance the risk of PTB. Moreover, African-American women have reduced access to efficacious health services, including contraception, control of hypertension and diabetes, preventive and therapeutic services for substance use, and mental health care, among others, that might address those risks (64–68). African Americans' lower access to and quality of medical care in general is well-documented (35–37). Screening and treatment for several conditions before pregnancy (e.g., nutritional deficiencies, STIs, hypothyroidism, hypertension, diabetes) have been associated with lower PTB rates (69–74), although some results are inconclusive (75, 76). Comprehensive preconception care programs have not, however, been associated with PTB reductions (77, 78). They typically begin only a few months before conception, however, which may be too late to mitigate risks accumulated across the life course (66). Although there is little research on whether racial disparities in preconception care contribute to disparities in PTB, it is plausible as a potential contributing factor.

### Elective and Indicated Cesarean Section

Although most deliveries are vaginal, medically indicated and elective cesarean sections before 37 weeks gestation increase the PTB rate. Cesarean delivery rates are higher in Black women than Whites (35.9 and 30.7%, respectively, in 2019) (16) independent of known risk factors (79), demographic characteristics (80), and differences in labor management (81). The Black/White gap in early elective cesareans (<39 weeks) closed in Oregon after the procedure was banned statewide (82). While there is insufficient research on the extent to which disparities in cesarean rates contribute to disparities in PTB, cesarean section is plausible as a potential contributing factor. Since most women deliver vaginally, however, disparities in cesarean section would likely make a limited contribution to disparities in PTB.

### Substance Use Disorders

Substance use before and during pregnancy has been linked repeatedly to adverse birth outcomes, including PTB (83–86). Racial differences in prevalence of substance use disorders, however, have been inconsistent, varying by substance, age, and sample. A nationally representative study estimated that binge-drinking-related PTB was considerably higher among White than Black women (87). At least before age 30, Black women of reproductive age are *less* likely than their White counterparts to smoke, engage in heavy drinking, or use marijuana (88–90) or benzodiazepines (91, 92) also found higher maternal use of alcohol and tobacco among Whites, but higher use of illicit drugs among Black mothers. Although African Americans smoke less, they are more likely to die of smoking-related causes than Whites (93). Theories explaining this paradox include: greater exposure to environmental toxins that potentiate smoking's adverse effects, melanin-related differences in nicotine metabolism, higher use of menthol cigarettes, and/or greater smoke intake per cigarette (93). To our knowledge, no published studies indicate that substance use explains the Black-White disparity in PTB.

### Diet/Nutrition

Nutritional deficiencies (e.g., iron, folic acid, zinc, vitamin D, calcium, magnesium, and imbalances of ω-3 and ω-6 polyunsaturated fatty acids) are more prevalent in Black vs. White women (94, 95), despite similar prenatal vitamin use (96), and have been associated with PTB (97, 98) and bacterial vaginosis, a PTB risk factor (94, 97). Pregnant Black women have higher intake of calories, fats, carbohydrates, and sugar, higher obesity rates, and inadequate intake of nutrient-dense foods (99–101), all of which are associated with PTB (102–105). It is plausible that nutritional disparities might contribute to the PTB disparity, but more robust studies are needed.

### Gestational Weight Gain (GWG)

In theory, excessive weight gain could increase PTB risk by increasing risk of hypertensive disorders (106, 107). A 2012 review by Headen and colleagues concluded that Black women are more likely than White women to have inadequate GWG, but not more likely to have excessive weight gain (108, 109). A large systematic review found that both inadequate and excessive pregnancy weight gain according to current guidelines were associated with higher risk of a number of adverse pregnancy outcomes, however, they also found that excessive weight gain was associated with **lower** risk of PTB (110). Analysis of data on 7,841 singleton births to U.S. Black women found that while high (>1.5 pounds/week) GWG was not associated with PTB among normal weight women, it was associated with increased risk of both spontaneous and medically indicated PTB among overweight and obese women (111). Leonard et al. (112) studied records of over 10 million non-Latino Black and non-Latino White women in the U.S. and concluded that moderate GWG was associated with reduced risk of PTB, especially the risk of early PTB among Black women. It is unclear whether excessive or inadequate weight gain contribute to the Black-White disparity in PTB.

### Obesity

Excessive adipose tissue is thought to trigger inflammation and immunological changes that could lead to PTB (113). Although a 2010 meta-analysis found that overweight, obese, and normal-weight women experienced similar PTB rates (114), examinations by PTB subtype indicate that overweight/obese women experience significantly more both medically-indicated (114) and spontaneous PTB (114–116), findings are inconsistent, however (113, 117). PTB risk could increase at higher BMIs because medical risk increases (e.g., diabetes, hypertension, pre-eclampsia, lipid-induced inflammation) (107, 113, 118). Black women's significantly higher prevalence of obesity (112) (see section Nutrition) makes obesity a plausible contributor to the PTB disparity but does not explain the disparity among non-obese women.

### Inter-pregnancy Intervals

Short (<12 months), very short (<6 months), and long (≥120 months) inter-pregnancy intervals (IPIs) are associated with increased odds of PTB (119–122). An IPI of 18 to 23 months has the lowest PTB odds for both Black and White women (122, 123). Black women are more likely to have suboptimal IPIs (122, 124, 125), although White women have significantly shorter median IPIs (26 months for White women vs. 30 months for Black women) (126). Hogue et al. (124) concluded that 4% of the Black-White PTB disparity could be explained by IPIs <6 months, which occur in approximately 10% of Black vs. 5% of White pregnancies. Short IPIs could contribute to the racial disparity but are unlikely to be a major cause given these findings.

### Age

Age differences do not explain the PTB disparity. The racial gap in PTB is evident across all childbearing age groups (127). While Black women have higher rates of teen births than Whites, Black PTB rates are lowest among women 15–19 and steadily increase with age. Geronimus (128, 129) found, net of confounders, that LBW, VLBW, and infant mortality increased with age in Blacks but not Whites, attributing this to “weathering,” the biological result of cumulative social disadvantage, it is plausible that weathering also affects PTB. Paternal age, birthweight (130), and gestational age (131) also have been related to PTB, which could reflect biological and/or social factors.

### Gestational Diabetes (GDM)

Both gestational (GDM) and chronic diabetes can lead to PTB by increasing risk of preeclampsia, which often results in preterm cesarean delivery (106, 107). GDM, although strongly associated with obesity, which is more prevalent among Black women, occurs less in Black women than in White women (112), prevalence estimates range from 4 to 4.9% and 4.5 to 7.0% among African American and White women, respectively (132–134). Odds of PTB secondary to GDM, however, are higher (aOR 1.56, 95% confidence interval 1.33–1.83) in Black women than White women (135). GDM may contribute to the PTB disparity, its lower prevalence among Black women is counterbalanced by a stronger effect size, at least in one study.

### Hypertensive Disorders of Pregnancy (E.g., Gestational/Pregnancy-Associated/Pregnancy-Induced Hypertension, Preeclampsia, Eclampsia)

Hypertensive diseases of pregnancy are not uncommon complications of pregnancy (136), occurring in 6–8% of all pregnancies overall (137). Hypertensive disorders (136), have been linked repeatedly with PTB (138–141). Hypertensive disorders occur more frequently and are more severe and more likely to result in PTB in Black than White women, controlling for pre-pregnancy hypertension (137, 142–145), possibly due to unmeasured comorbidities (146, 147). A population-based longitudinal study of 2.5 million non-diabetic New York births found rates of preeclampsia of 3.2 per 100 hospitalizations among Black women and 1.8 per 100 hospitalizations among White women (137). Inflammation, thought to play an important role in PTB, may play a role in both pre-pregnancy hypertension and pregnancy-induced hypertensive disorders (148, 149). Hypertensive disorders of pregnancy likely contribute to the Black-White disparity in PTB, but the underlying causes that trigger these disorders require further elucidation.

### Pre-pregnancy (Pre-existing) Diabetes

Pre-pregnancy Type I and II diabetes have been consistently associated with PTB (150–154), although the risk appears higher in women with Type II diabetes (155), who may experience other associated risks (e.g., higher weight and age, less prenatal care and glycemic control) (156). Compared with White women, Black women develop both Type I and II diabetes at higher rates and younger ages (157), and receive inferior clinical care (158). The age-standardized rate of pre-pregnancy diabetes among women giving birth in 2010 was 0.26 per 100 births among Black women and 0.16 per 100 births among non-Hispanic White women (159). Black women have worse outcomes (e.g., end-stage renal disease) than otherwise similar White women (160). Pre-pregnancy diabetes disparities may play a role in PTB disparities but the relatively low prevalence makes it unlikely to explain a major part of the disparity, rising rates over time, particularly among Black women, however, should be cause for concern (159).

### Pre-pregnancy (Chronic) Hypertension

Pre-pregnancy hypertension has been linked consistently to pre-eclampsia (161–163), gestational and pre-existing diabetes (161, 164, 165), and PTB (43, 163, 166, 167). Although good blood pressure control during the first trimester can reduce a woman's risk of PTB (168), Black women not only have higher rates of pre-pregnancy and pregnancy-related hypertension than White women (169), they also appear more susceptible to their adverse effects on gestational length (170). Estimates of the prevalence of pre-pregnancy hypertension using 2011–2016 NHANES data are 18.7% among Black women and 8.2% among White women (171). Pre-pregnancy hypertension is a highly plausible contributor to the Black-White disparity in PTB.

### Infection

PTB is strongly associated with periodontal and urogenital infections [e.g., bacterial vaginosis (BV)] (19, 172–180). Black women have higher prenatal infection rates (176, 177, 181–183). They have greater biological (e.g., absence of *Lactobacillus* ssp and high levels of *Gardnerella* ssp and *Mobiluncus* ssp), social (e.g., socioeconomic disadvantage) and psychosocial [e.g., stress (180, 181)] risk factors. A study using 2001–2004 NHANES data found a prevalence of Bacterial vaginosis of 51.4 and 23.2% among Black and White women, respectively (184). When infected, Black women may be more susceptible to PTB than White women (185).

Infection is a biologically plausible contributor to the Black-White disparity in PTB. Lack of consistent improvement in PTB with treatment, however (181, 186–188), have been attributed by some to inadequate treatment (182, 188), or our inadequate knowledge of pathogens (178). This raises the question whether infection is a cause of PTB, a marker, or an intermediate outcome of some underlying factor, such as inflammation or stress-induced immunocompromise (174, 187).

### Microbiota

Microbiota are microorganisms, beneficial and harmful, living in or on the body, the microbiome is the collective genome of these microorganisms (189). The vaginal microbiota of White and Black women differ significantly (190–192). White women are more likely to have microbes that promote immunity and decrease PTB risk (e.g., BVAB3, *L. crispatus, L. jensenii, L. gasseri*), Black women have a higher prevalence of vaginal microbes associated with PTB (e.g., *G. vaginalis*, BVAB1, BVAB2, Atopobium vaginae, Megasphera, Sneathia, and Prevotella) (181, 190, 193, 194), although not all studies have reported this (195). Black women's higher douching rates could contribute to racial differences in vaginal microbiota (190), which might help to explain PTB disparities, as some studies have linked douching to PTB or PTB risk factors (e.g., urogenital infection) (180, 196–199). A person's microbiota also may be altered by oral infections (200) (see section Infection), and infections from gut-associated bacteria secondary to premature rupture of membranes (201) or inflammatory bowel disease (202), both of which have been associated with PTB (203–205).

### Neighborhood Environmental Exposures

Racial residential segregation, a consequence of racism at structural and interpersonal levels, is associated not only with social and economic disadvantage, but also environmental toxicity (206–209). Black people are far more likely than Whites to live in racially segregated neighborhoods (210, 211), regardless of level of income, residential preferences, and housing affordability (212, 213). Because of environmental injustice, race/ethnicity is a stronger predictor of residence in polluted areas than low educational attainment (214), with Black people being significantly more likely to experience toxic exposures (e.g., air, traffic, water, industrial) (207, 214) linked to PTB (207, 215–218). Higher percentages of Black people in an area are associated with higher PTB rates among both Blacks and Whites, an effect that is not modified by area-level socioeconomic characteristics (219–225).

The combination of greater exposure to environmental toxins and to neighborhood social disadvantage might increase Black women's susceptibility to adverse birth outcomes (226). In a 2020 systematic review of 58 studies of air pollution and pregnancy, 84% of studies reported a significant association of air pollution with adverse birth outcomes, including PTB, with 50% also reporting significant disparities (227) (see section Neighborhood Social Disadvantage). Environmental hazards in neighborhoods are a highly plausible and potentially major contributor to the Black-White disparity in PTB.

### Cold and Heat

Pregnancy exposure to excessive cold and heat has been associated with PTB (228–233). African Americans experience greater heat exposure, due to lower access to in-home central air conditioning and cooled public spaces than Whites (234–236). Bekkar et al.'s (227) systematic review of pregnancy and heat studies found that 90% of studies reported a significant association of excessive heat with PTB and most reported racial disparities. Both heat (228, 237, 238) and cold (238) may differentially impact Blacks' vs. Whites' PTB risk. Differential exposure to heat could plausibly contribute to the PTB disparity, however, there are too few studies to determine the magnitude of the contribution.

### Genetic and Epigenetic Factors

Several observations indicate that genetic differences contribute to the risk of preterm birth (PTB). The likelihood of PTB is increased for women who were born preterm themselves, have a family history of PTB, or have had a prior PTB (239, 240). Family and twin studies have estimated the heritability of PTB—a measure of the relative contribution of genetics—to be in the range of 14–40% (239–241).

While these observations support inherited susceptibility to PTB, epigenetic effects may also play a role—that is, changes in gene expression as a result of environmentally mediated changes in the chromosomal matrix. Epigenetic changes at three genetic loci have been associated with risk of PTB (242–245). Social and environmental factors associated with PTB risk, such as diet and exposure to stress and environmental toxins, are potential mediators of epigenetic change. One recent study identified gene expression signatures associated with both vitamin D insufficiency (which is more common among Black women and is associated with PTB, as noted above under Diet/Nutrition) and PTB (246).

Molecular studies have identified specific inherited gene variants that are associated with a higher likelihood of PTB. However, large-scale studies have been required to reliably identify them, and the variants explain only a small proportion of the variability in PTB risk. For example, a genome-wide association study of 43,568 women of European ancestry identified and replicated variants in six genes associated with PTB risk (247, 248). The genes were involved in a range of functions, including uteroplacental circulation, development of the female reproductive system, cell energy and metabolism, adipocyte differentiation, and selenium metabolism. Although the findings were reproducible, the variants explained <1% of the variance in PTB. Other studies have implicated these and other functions, including endocrine, vascular and metabolic functions and inflammatory response, however, these studies have been limited by lack of replication or by inconsistent findings (239, 245, 249–251).

Given the documented importance of social and other environmental contributors to PTB risk, some research has focused on gene-environment interactions. A genome-wide association study of the Boston Birth Cohort, a longitudinal study of 1,733 African-American women, found an interaction between a variant in the *COL24A1* gene and body mass index. Although the variant did not predict PTB risk independently, the study found that normal weight women with the variant had increased risk, while overweight and obese women with the variant had decreased risk (252). In a second cohort evaluated as part of the same study, the finding was replicated among African-American women, but not among women of European ancestry. This finding suggests that the effect of the *COL24A1* variant on PTB risk is modified by exposures associated with weight status in African-American women, the negative result in European-American women could reflect differences in weight-associated exposures between African- and European-American women. Other potential interactions between genetic risk and environmental exposures have also been identified to play a role in causing spontaneous PTB, including smoking (253, 254) and bacterial vaginosis (255). The positive selection of variants in the progesterone receptor (*PGR*) gene observed in East Asian populations has been hypothesized to result from reduced risk of PTB associated with such variants (256). Additionally, while genetic variation in the microbiome may play a role in PTB risk, “environmental exposure factors cannot be excluded to play a role in the shaping of the cervicovaginal environment and the risk to… [spontaneous PTB]” (257).

Most research has focused on genetic susceptibility to PTB in women. The situation, however, is undoubtedly more complex, with contributions from fetal genetics (253, 258) and potential for gene-gene and gene-environmental interactions involving both fetal and maternal genomes. In addition, physiological studies suggest more than one pathway to PTB, genetic contributors to PTB and associated gene-gene and gene-environment interactions may vary for different pathways (259–261). Systems biology approaches incorporating studies of gene expression, the proteome and metabolome (262), and utilizing innovative approaches to the molecular study of pregnancy over time (263) are likely to be required to elucidate different genetic and environmental contributors to PTB. While maternal influences in predicting gestational length appear stronger, for example, indicating 1.22 additional days at birth for each additional week of the mother's gestational age, the father's gestational age has also been shown to be associated with PTB (131).

Genetic influences are a plausible contributor to the racial disparity in PTB, the magnitude of the effect, however, is likely to be small. Epigenetic effects—triggered by, e.g., environmental exposures and chronic stress associated with socioeconomic hardship and discrimination—are highly plausible as a potentially major contributor to the disparity.

**MIDSTREAM FACTORS** are hypothesized to affect PTB through their influence on downstream factors.

### Stress

Psychosocial stress (stress) involves life demands (stressors) that strain or exceed adaptive resources, resulting in bio-psycho-social responses (the stress response) downstream that could compromise health (264, 265). Considerable research links stress to PTB, likely through stress-induced physiological mechanisms (266–269) including inflammation, immune dysregulation (270), and effects on behaviors (271, 272). Considering how stress could influence PTB by influencing behaviors, almost all of the plausible downstream factors examined here have potentially substantial behavioral influences, e.g., preconception care, nutrition, infection, microbiota, obesity (behavioral risk factors for and self-management of), pre-pregnancy hypertension, diabetes, hypertensive disorders of pregnancy, or gestational diabetes, gestational weight gain, and inter-pregnancy intervals. Heat/cold and genetics are the exceptions, however, epigenesis could have strong behavioral influences.

Although conclusions are not definitive in every case, studies have linked stress, directly or indirectly, to many of the plausible downstream factors, including: diet/nutrition (273), infection (269), microbiota (274, 275), obesity (276), pre-pregnancy hypertension (277), hypertensive disorders of pregnancy (278), pre-pregnancy diabetes (279), gestational weight gain (280), gestational diabetes (281), and epigenetic effects (282) (The many studies linking stress to the racial disparity in PTB are noted in the last sentence of the final paragraph in this section on stress).

Examining one downstream factor in more detail as an example, hypertension is a downstream risk factor plausibly influenced by stress, although further study is needed. Stress is known to induce temporary vasoconstriction and subsequent elevated blood pressure, however, the contribution of stress to the development of hypertension remains equivocal due to methodological inconsistencies (277, 283–285). Inflammation, which can result from chronic stress-related immune dysregulation (270), has repeatedly been linked to hypertension in both pregnant and non-pregnant humans (286–288). Inflammation is thought to be involved in hypertensive disorders of pregnancy (148, 149), preeclampsia itself has been considered “an excessive maternal inflammatory response to pregnancy” (289). A recent study in the Netherlands found elevated hair cortisol levels (measured from 3 months preconception to the end of the 2nd trimester) and anxiety scores (at hospital admission) in women with preeclampsia (290). A systematic review concluded that chronic stress is more likely than acute stress to result in prolonged elevated blood pressure (285).

Although Black women report more stress (291–293), this has not consistently explained PTB disparities (294–296), possibly due to inadequate measurement (297, 298), particularly a tendency to focus exclusively on stress during pregnancy. The physiologic “wear-and-tear” caused by chronic stress can compromise women's reproductive health well before they become pregnant (299). However, few studies consider life-course exposure to chronic social stressors (such as racism and/or its effects, including economic hardship), which may better explain PTB disparities than stress only during pregnancy. There is broad scientific consensus about an important role for life-course stress in the PTB disparity (66, 129, 300–302), including its biological plausibility (303, 304). Stress is a highly plausible, potentially major contributor to the racial disparity in PTB.

### Depression

Depression has been associated repeatedly with PTB (305–307). Black people, however, are less likely to have a major depressive disorder diagnosis, despite higher self-reported psychological distress (308). While differences in symptomatology or reporting may explain this paradox, the literature indicates that depression does not explain PTB disparities.

### Resilience

The literature on maternal resilience (as reflected by, e.g., optimism, self-efficacy, self-esteem) and PTB is inconsistent. Some studies report significant associations (309–312), but not all do (313). Others report paradoxical associations such as greater optimism among African-American women who deliver preterm (314). Wheeler et al. (315) reported a stronger positive relationship between self-efficacy and PTB in Black vs. White women. Evidence is insufficient to draw conclusions about the role in the PTB disparity of factors associated with resilience.

### Coping

Research suggests that passive/avoidance coping may increase African Americans' PTB risk (314, 316), while active coping may moderate effects of stress (e.g., due to racial discrimination) on PTB (317). African Americans reportedly engage in less active (318) and more passive/avoidance coping (319) than Whites or Hispanics, particularly when facing racism (320). Given limited research on how coping could moderate stress effects in pregnancy (266, 321), coping differences might plausibly contribute to PTB disparities, the relevant literature, however, is inconsistent and insufficient.

### Social Support

Extensive research indicates that social support can buffer the negative effects of stress, thereby protecting against poor health outcomes (322–330), including PTB (331, 332). Greater support has been associated with less inflammation (314) and less “weathering” (chronic stress-related biological aging) in Black people specifically (333). Lower support has been linked to shorter telomeres (an indicator of cellular aging) in perinatal women (334). Social support has been observed to be beneficial for White women with low but not high levels of stress. Among Black women, however, social support seems protective regardless of stress level (335). Many Black people draw substantial social support and health benefits from religious involvement (333, 336), and spirituality and family/friend support have been shown to predict pregnant women's anxiety, depression, and stress (337). Black people report fewer friends than Whites, but do not differ in feeling adequately supported (338). Findings on racial differences in family support vary (338, 339). It is plausible in theory that social support could contribute to the Black-White disparity in PTB. Given the inconsistencies in findings and the literature's focus only on support during pregnancy, however, it is not possible to assess the contribution of social support to Black-White disparities in PTB.

### Midstream Paternal Factors

Midstream paternal factors (Note: paternal genetic factors are discussed in the section on genetic factors, and paternal age is noted among downstream factors). Midstream factors influence downstream factors and in turn are influenced by upstream factors.

Research indicates that fathers' social characteristics can influence PTB risk. Paternal hazardous occupational exposures (lead, x-rays), and age and birthweight (considered downstream factors in this paper) have been associated with PTB, although research is limited and results are inconsistent (130). Paternal education and lifetime socioeconomic factors predict PTB, independent of maternal demographics (130, 340, 341), and paternal involvement accounts for more of the racial disparity in PTB than maternal education (43). Paternal support predicts lower levels of anxiety, depression, and smoking in pregnancy (342), whereas, paternal prenatal depression, which may inhibit paternal support, predicts PTB (343). Partner-related stressors, such as separation/divorce, death, and interpersonal violence are higher at lower socioeconomic levels (344) and increase PTB risk (345–347). Although Black people are more likely to report such exposures (348), racial differences in stressful events appear to contribute little to PTB disparities (296). Theoretically, Black men's disproportionate incarceration rates could help to explain the Black-White PTB gap through various economic (349), behavioral (350), psychosocial and physical health pathways (351). Thus, it is plausible that paternal factors could contribute to the Black-White disparity in PTB through multiple causal pathways, including those involving stress and socioeconomic factors.

### Socioeconomic Factors

A range of socioeconomic factors—including but not limited to income, educational quantity and quality, wealth, childhood/lifelong socioeconomic conditions, and neighborhood socioeconomic conditions—could plausibly affect PTB rates and the racial disparity, by affecting downstream factors. Research has linked one or more socioeconomic factors with most of the downstream factors, e.g., preconception care (352), cesarean section rates (353), infection (354), nutrition (355), alterations in microbiota (356, 357), obesity (358), pre-pregnancy hypertension (359, 360), pre-pregnancy diabetes (361, 362), hypertensive disorders of pregnancy (145, 363), gestational diabetes (364, 365), suboptimal gestational weight gain (366), interpregnancy intervals (367), and both of the unlikely factors, standard prenatal care (368) and substance use disorders (369, 370). Below we briefly summarize knowledge of the role of several different socioeconomic factors in PTB.

### Income and Education

Black people have lower levels of income and education than Whites, and earn less for similar levels of education (371), reflecting centuries of discriminatory policies and practices (e.g., slavery, Jim Crow, redlining, segregation, mass incarceration) that have limited their socioeconomic opportunities (371–377). However, the role of socioeconomic factors in PTB disparities is not simple. Many studies have observed that differentials by race (332) and nativity (immigrant vs. US-born Black women) (378–381) persist after controlling for income and/or education. Income and education consistently predict White, but not Black, PTB rates, with the racial gap widest at the highest socioeconomic levels (382–385), and little (316, 386) to no (383, 387) racial differences in PTB rates among socioeconomically disadvantaged women. Income predicts exposure to environmental hazards and lack of exposure to health-promoting conditions in the home and neighborhood. Income, for example, is likely a major determinant of access to air conditioning, this is consistent with African Americans' markedly lower access to air conditioning (235), which could reduce the PTB risk associated with excessive heat exposure (227).

### Childhood and Lifelong Socioeconomic Status (SES)

Although rarely considered, both classic (388, 389) and more contemporary studies, e.g., Dominguez et al. (300), demonstrate that childhood rather than current SES is a stronger predictor of birth outcomes. Socioeconomic disadvantage in childhood is more likely among Blacks than Whites (390), even among those of similar adult income or education (300, 391). Childhood adversity has been associated with PTB (392–394). Childhood disadvantage could affect a woman's risk of PTB in multiple ways (300, 395), including exposures to air pollution and other toxins, inadequate nutrition, and chronic stress (see Environmental Hazards and Stress sections).

By contrast, upward mobility (396), intergenerational high SES (397), and lifelong residence in high-income neighborhoods (398) are associated with better African American birth outcomes and/or smaller racial disparities, adjusting for confounders. However, upward mobility's benefits appear stronger in White mothers (397, 399, 400), this may reflect the chronic stressors that many upwardly mobile African-American women face (383, 395, 401–403).

### Wealth

Wealth, the value of net assets, is a better indicator of economic security and stability than more volatile factors, such as income (404). For a given level of income and/or education, Blacks have accumulated far less wealth than similar Whites (see Racism section) (371, 376, 404, 405). Greater wealth across the life course could reduce PTB risk by reducing adverse exposures, increasing health protections, and mitigating chronic stress (66). We could not identify empiric research on wealth as a causal factor in PTB.

### Educational Quality

Researchers frequently assess educational attainment (years and/or degrees), but rarely consider educational quality, which could be an important socioeconomic indicator of PTB risk. Black children are more likely than Whites to attend under-funded, lower-quality schools without advanced coursework, and to be corporally punished or suspended/expelled, all of which puts them at a disadvantage for college and the labor market (406–409). Although racial disparities in college/university enrollment have narrowed, Blacks are significantly less likely than Whites to graduate with a bachelor's degree and attend prestigious institutions (410, 411). We could not identify empiric research on educational quality as a causal factor in PTB.

### Neighborhood Socioeconomic Disadvantage

Neighborhood socioeconomic disadvantage (e.g., high poverty, unemployment, racial segregation) has been repeatedly linked to PTB (412, 413). Biologically plausible pathways include stress—e.g., community violence (414)—and environmental exposures (e.g., polluted/toxic air, ground, water, housing) (see section Neighborhood Environmental Exposures). Black women are more likely to reside in disadvantaged neighborhoods, and, when doing so, are more vulnerable to PTB than their White neighbors (383, 412). Among higher-SES women, the racial disparity in LBW is lowest among those in racially congruent neighborhoods and widest among those in predominantly White neighborhoods (415).

### Socioeconomic Factors Are Highly Plausible Contributors to the Racial Disparity in PTB

No study to our knowledge has measured all of the socioeconomic factors that could plausibly affect PTB. Nevertheless, the persistence of racial differences net of SES measures, and the complex patterns of SES findings indicate that socioeconomic factors alone cannot explain racial disparities in PTB.

**UPSTREAM FACTORS** are hypothesized to influence PTB through their influence on midstream factors, which in turn influence downstream factors.

There is considerable scientific consensus that racism, operating through multiple pathways, is a plausible upstream cause of many Black-White health disparities (416, 417) including birth outcomes (332, 418, 419). Self-reported/perceived exposure to racism in childhood (300, 420), the perinatal period (302, 421), and across the life course (316, 317, 395, 402, 422–426), as well as worry about potential exposure to racism (395) have been associated directly or indirectly (427, 428), net of confounders, with a 1.5- to 3-fold increased risk of adverse African American birth outcomes (395, 421, 429) and with the Black-White disparity in PTB (395, 424). While not all studies have observed an association between racism and PTB—e.g., Lu and Chen (296), Murrell (430)—measurement has been highly variable (298, 431, 432). Most studies, for example, have examined racial discrimination only during pregnancy or the year before delivery, fewer (317, 395, 402, 424, 429) have examined lifetime experiences, which are more likely to influence PTB based on knowledge of stress physiology (270). Mustillo et al. (424) found that the Black-White disparity in PTB became non-significant after adjusting for lifetime experiences of racial discrimination, Braveman et al. (395) had similar findings on examining worry about discrimination. Diverse experiences of racism have been studied, from overt insults and other incidents of unfair treatment to more subtle experiences and pervasive worry or vigilance in anticipation of experiencing discrimination. At the structural level, a recent study found that African American women's residence in redlined neighborhoods was associated with an increased risk of PTB (433) [Redlining is the practice of denying (or charging more for) services, such as bank loans and insurance, to residents in racially segregated areas, red lines were drawn on maps to mark areas where loans would not be given, which corresponded strongly to areas where many African Americans live].

**Figure 2:**
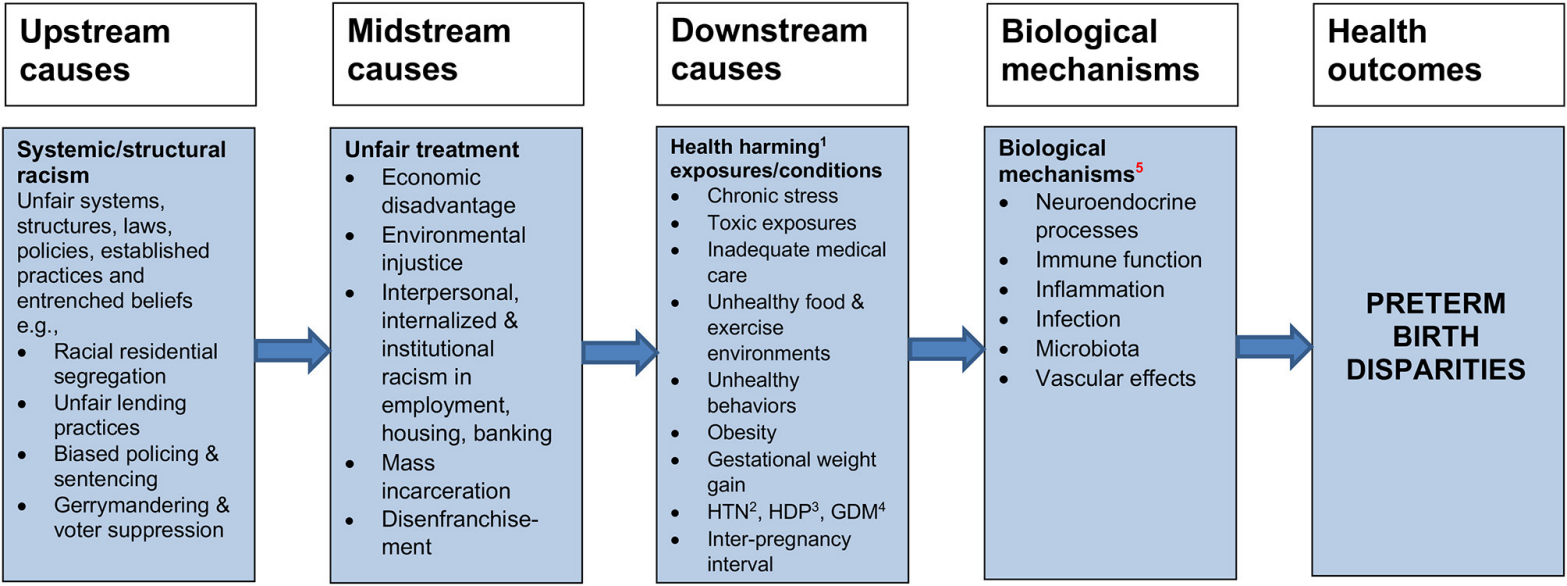
Racism plausibly may affect PTB as an upstream factor, acting through midstream and downstream factors that more directly trigger the physiologic mechanisms. 1. Health harming or lack of health-promoting exposures/conditions. 2. HTN: Hypertension. 3. HDP: Hypertensive disorders of pregnancy. 4. GDM: Gestational diabetes mellitus. 5. Epigenetic effects are not displayed because they may occur through exposures at each step along the causal pathways.

Figure 2 is a simplified schematic representation of how racism could increase risks of PTB through a range of biologically plausible pathways previously noted in this paper, operating through midstream factors such as stress and socioeconomic disadvantage, which in turn influence downstream factors that directly activate physiologic mechanisms. Racism is a pervasive system of unequal power relationships, based on ideological notions of the inherent superiority of Whites and inferiority of People of Color (POC), it unfairly advantages Whites and marginalizes POC.

Racism is a chronic stressor (434–436) that could affect PTB through the body's physiologic response to stress (e.g., neuroendocrine, immune, inflammatory, and vascular mechanisms) (268, 270, 437). Racism operates not only in interpersonal interactions but also structurally via laws, policies, institutions, and practices, which historically produced and continue to perpetuate systematic disadvantage affecting POC including African Americans, examples include discriminatory lending practices and residential segregation, which produce educational and economic disadvantage, and hazardous environmental exposures. These practices perpetuate racial disparities in health and in opportunities to be healthy, even when, arguably, there is considerably less conscious intent to discriminate today than when major civil rights legislation was enacted in the 1960s.

Racial residential segregation is a salient example of structural racism, it tracks low-income and many middle-income African Americans into neighborhoods that are unhealthier in multiple ways, including exposure to environmental hazards, inferior schools, lack of employment opportunities, poor services, concentrated poverty and the accompanying sources of chronic stress (438). All of these could increase risks of PTB through a range of pathways noted in this paper. Racial segregation produces and perpetuates racial disparities in income and wealth, for example, through inferior educational and occupational opportunities, redlining, and other discriminatory banking/lending practices that make it far more difficult to own a home or to start, sustain, or expand a business (438). Discrimination in hiring, promotions, and pay also constrain income and wealth. The resultant socioeconomic disadvantage from all these sources constrains the options one has for housing and neighborhoods, and for access to health care, nutritious food, and safe green space for exercise and leisure activities, this disadvantage produces chronic stress due to constantly facing daily challenges to meet one's family's basic needs with inadequate financial resources (see Socioeconomic Factors section).

## Discussion

This literature review confirms the widely held scientific view that the causes of the Black-White disparity in PTB, and the causes of PTB itself, are largely unknown. Current knowledge, however, identifies many plausible—and some implausible—potential causes, all of which warrant further research. This review also confirms that the etiology is likely to be both multifactorial and complex, which may partly reflect that PTB is not a single disease but several distinct clinical entities with varying etiologies.

While definitive proof is lacking for most hypothesized causes, most of the causes we considered are plausible—including biologically plausible—as potentially important contributors to the disparity. Most of the downstream factors and all of the midstream and upstream factors examined are plausible. Standard prenatal care and substance abuse disorders appear less likely as causes of the Black-White disparity in PTB, based on the empiric evidence to date. Despite well-documented racial and socioeconomic disparities in care, the weight of evidence has not supported lack of standard prenatal care as a cause of the PTB disparity, disparities in preconception care and in cesarean section rates, however, are plausible contributors.

While it is plausible that differences in nutrition, infections, and microbiota could potentially contribute to—and preconception care and/or group prenatal care might diminish—the Black-White disparity in PTB, evidence is limited on nutritional deficiencies and preconception care and inconsistent on infections, microbiota, and group prenatal care. Excessive gestational weight gain, gestational diabetes, hypertensive disorders of pregnancy, short inter-pregnancy intervals, and pre-pregnancy obesity, diabetes, and hypertension are plausible but cannot explain the PTB disparity among women without these conditions.

PTB has consistently been linked with exposures to toxic physical hazards, such as air pollution. Socioeconomic disadvantage is not the only cause of disproportionate exposure to hazardous neighborhood conditions. Woodruff et al. (214) found that race was a stronger predictor of residence in polluted areas than educational attainment (which is strongly correlated with income). Environmental injustice—the disproportionate location of toxic substances in Black and Brown communities—is a highly plausible contributor to the Black-White disparity in PTB. Many Black women even of relatively high SES live in racially segregated areas (439, 440) with hazardous exposures, which could contribute to the lack of expected improvement in PTB among higher-SES Black women.

Along with disparities in prenatal care and substance use, genetic differences have often been hypothesized to explain the racial disparity in PTB. When considering the potential role of genetic factors, it is important to note scientific consensus that race is primarily a social, not a biological, construct (441). Genetic variation is seen both within and across different ancestral populations. However, racially defined populations in the United States tend to demonstrate patterns of genetic variation associated with continental ancestry. Thus, the prevalence of many monogenic disorders, such as sickle cell disease and cystic fibrosis, varies among Black and White U.S. populations. If genetic research had identified common gene variants with a large independent impact on PTB risk, and if such variants differed in prevalence across racial populations, one might expect a significant genetic contribution to racial differences in PTB. However, large-scale genomic studies have failed to identify such variants. Current evidence indicates that the genetic contribution to PTB risk occurs via the aggregate effect of multiple variants, each of small effect, often involving gene-environment (and likely gene-gene) interactions. Furthermore, environmental exposures may trigger epigenetic change, environmental risks include factors as disparate as diet and smoking, the microbiome, social exposures such as racism and poverty, and, related to racism and poverty, physical exposures to toxins and air pollution (442). Even without controlling for epigenetic effects, heritability estimates suggest that the environment plays a greater role than genetics in PTB risk (443). Due to a long history of racism, exposure to most of the environmental risks associated with PTB differs markedly among U.S. Black and White populations. Furthermore, the favorable birth outcomes of Black African immigrants to the U.S. do not support a genetic basis for the PTB disparity between US-born Black and White women. If genetic differences were the basis, one would expect Black African immigrants to the United States to have PTB rates at least as unfavorable as those of US-born Black women, whereas Black African immigrants' PTB rates are similar to those of U.S. White women (444). Based on all these considerations, genetic factors contribute to PTB risk but are likely to explain at most a small fraction of the Black-White difference in PTB. Gene-environment interaction studies may help to better define PTB subtypes and their biological mechanisms, potentially informing preventive and therapeutic interventions, this should be a high priority for further research.

While many hypothesized causes are plausible, no single downstream factor explains the magnitude of the disparity, most are likely to play a small role. This suggests that PTB may reflect the combined effects of multiple causes and pathways, and that we must identify the upstream factors that initiate the harmful pathways: the causes of the causes. Identifying downstream causes can be crucial to mitigate downstream health damage and at times may help identify upstream causes. It generally does not, however, answer the question: What initiated the causal chain? What upstream forces produced these downstream conditions? Answering this is vital for prevention. Actions targeting the final steps in long, complex causal chains may be ineffective without addressing the underlying causes that initiated the chains. Furthermore, the downstream factors cannot explain the strong social patterning of the racial disparity in PTB, for example, the absence of a PTB disparity among Black African immigrants compared with U.S.-born White women.

### Considering the Causes of the Causes

For example, consider racial disparities in nutrition, obesity, and environmental toxins as potential downstream causes of the racial disparity in PTB. Racial residential segregation makes Black women of both moderate and lower incomes substantially more likely to reside in areas with many fast-food outlets and convenience stores (445, 446), which are associated with obesity and poor nutrition (446, 447), and in areas less conducive to exercise, an important determinant of obesity. Black women are likely to experience chronic stress associated with racial discrimination (300, 395, 448) as well as economic disadvantage. At the same level of education, Black women have less income, at the same income level they have less wealth, coping with low financial resources is stressful. Chronic stress has been linked with less healthy behaviors in general (449) and specifically to higher consumption of and potential addiction to high-fat, high-sugar foods, leading to obesity and other metabolic diseases (450–452). Overconsumption of high-fat, high-sugar foods with little nutritious value is often accompanied by under-consumption of more nutritious foods, which increases risk of nutritional deficiencies (453, 454). Racial segregation combined with environmental injustice also means that Black women are more likely to be exposed to environmental toxins.

The literature documents several upstream/midstream causes of the downstream causes. Considerable empiric research has linked each hypothesized midstream cause (except depression, which has inconsistent results) with the racial disparity in PTB, through their influence on one or more downstream factors. Stress could affect PTB by activating neuroendocrine and immune mechanisms leading to inflammation and immune system dysfunction known to cause PTB. Stress could alter a woman's microbiota, her immune response to infection, and her susceptibility to chronic disease including pre-pregnancy conditions. Stress could trigger epigenetic changes influencing the risk of PTB.

Extensive literature also documents stress as a strong influence on behaviors, and most of the downstream factors have substantial behavioral aspects. Depression, resilience, coping, and social support are other midstream factors that could plausibly influence many downstream causes by influencing behaviors, however, research linking them with PTB is insufficient. Also based on the broader health literature rather than extensive empiric studies of PTB, it is plausible that psychosocial factors associated with resilience, coping, and social support could modify the effects of chronic stress on PTB.

### Socioeconomic Disadvantage Across the Life Course

Extensive research documents pervasive socioeconomic disadvantage among Black women compared with White women, with respect to socioeconomic factors including income, education, and neighborhood conditions, and for lifetime experiences of these factors. These socioeconomic factors have been linked with PTB (383) and/or multiple downstream factors. Socioeconomic factors alone, however, cannot explain the social patterning of PTB disparities, particularly the higher than expected PTB rates among socioeconomically-better-off Black women. Forces farther upstream have produced Black women's socioeconomic disadvantage.

### Looking Farther Upstream: Racism

Racism is the only factor identified by this review that directly or indirectly could explain the racial distributions of all of the downstream and midstream causes, including socioeconomic factors. Racism as a fundamental, upstream cause is a highly plausible major contributor to the Black-White disparity in PTB through multiple different causal pathways and biological mechanisms. Racism explains the racial disparity in socioeconomic factors—the legacy of slavery, 100 years of Jim Crow laws, racial residential segregation, and ongoing discrimination in employment, housing, policing, and sentencing. All of these have relentlessly deprived African Americans of socioeconomic opportunity. Socioeconomic disadvantage has differentially exposed African Americans to the chronic stress that accompanies facing daily challenges, such as childcare and feeding and sheltering one's family, with inadequate financial resources. Racial segregation has placed Black women in pervasively stressful neighborhood surroundings (often characterized by fear, deprivation, despair, and/or crime), it has systematically exposed them to environmental toxins, including air pollution. Racial discrimination is a powerful direct source of stress, considering both overt incidents and the pervasive vigilance one needs to be prepared for potential incidents affecting oneself or loved ones (455). Racism could differentially threaten or undermine resilience and coping among Black compared with White women, increasing vulnerability to the harmful effects of chronic stress.

Racism may also help to explain some of the social patterning of PTB. For example, favorable PTB rates among Black immigrants from Africa could conceivably be explained by immigrants' lack of exposure to racial discrimination earlier in their lives, during sensitive developmental periods, with its long-lasting neuroendocrine and immune sequelae. The higher than expected PTB rates among socioeconomically better-off Black women reflect the fact that even middle-class Black women often live in segregated neighborhoods, where they are exposed to the same environmental hazards and poor resources as their socioeconomically worse-off counterparts. It also could reflect cumulative physiologic wear and tear due to the chronic stress, over their lifetimes, associated with overcoming barriers to educational and occupational success. Some have hypothesized that it also may be due to higher-SES Black women experiencing more discrimination at work because of being the only or among few Black women at their professional level (383, 395, 402), this possibility is supported by studies showing that higher-income/education Black women report more discrimination than their lower-income/education Black peers (395, 456).

All of the plausible causal factors considered in this study deserve further research, especially those likely to affect many women or have large effects. More priority should be given to rigorously studying the upstream/midstream factors that are highly plausible and may present opportunities to prevent multiple destructive pathways from being set in motion. Some potential causes—such as racism, environmental injustice, and lifetime socioeconomic disadvantage—are so plausible, inter-related and amenable to policy intervention, and have such compelling reasons to address them, that they deserve particular attention. A compelling pragmatic reason to address racism, environmental injustice, and socioeconomic disadvantage is the extensive evidence that they are upstream causes for many other adverse health outcomes in addition to PTB. Another compelling reason is ethical values, which require that we not only mitigate the end-organ damage caused by unfavorable downstream factors, but also address the inequitable midstream and upstream conditions that produce downstream bodily harm through multiple pathways. While much is unknown, both existing knowledge and core ethical values can and should guide policies and research agendas.

## Author Contributions

PB, TD, and WB drafted the initial manuscript, with contributions from JWC, FJ, TH, JA, PW, DS, and GS. TD and PB revised the manuscript with input from the other authors. All of the authors participated in conceptualizing the study, in reviewing drafts, and in reviewing and approving the final manuscript.

## Conflict of Interest

FJ is President of Majaica, LLC. CDC funds supported March of Dime's (MOD's) Prematurity Campaign, which included convening a scientific workgroup. CDC funds were not used to directly support development of this manuscript. MOD funded a small stipend for TD to conduct a comprehensive scientific edit of the initial consensus statement to prepare it as a manuscript of publishable length. MOD funded a small stipend for TH for additional assistance with references. The remaining authors declare that the research was conducted in the absence of any commercial or financial relationships that could be construed as a potential conflict of interest.

## Publisher's Note

All claims expressed in this article are solely those of the authors and do not necessarily represent those of their affiliated organizations, or those of the publisher, the editors and the reviewers. Any product that may be evaluated in this article, or claim that may be made by its manufacturer, is not guaranteed or endorsed by the publisher.
